# Drei kulturelle und organisatorische Welten

**DOI:** 10.1007/s11613-021-00727-2

**Published:** 2021-11-03

**Authors:** Larry Hirschhorn, Thomas Giernalczyk, Martin Holle, Mathias Lohmer, Markus Zimmermann

**Affiliations:** 1 Park Square, 31 Saint James Avenue, Suite 720, 02116 Boston, MA USA; 2 M19 – Manufaktur für Organisationsberatung GmbH, Universität der Bundeswehr München, Bauerstraße 19, 80796 München, Deutschland; 3 Grünenthal 30, 52072 Aachen, Deutschland; 4 Feilitzschstraße 36, 80802 München, Deutschland; 5 Bauerstraße 19, 80796 München, Deutschland

**Keywords:** Covid-19, Virtuelle Zusammenarbeit, Bürokratische Welt, Projektwelt, Transaktions-Welt, Personalentwicklung, Covid-19, Virtual collaboration, Bureaucratic world, Project world, Transaction, Corporate design, Human resources

## Abstract

Covid-19 beschleunigt virtuelle Formen der weltweiten Zusammenarbeit. Das hat weitreichende Konsequenzen für Struktur und Prozesse von Organisationen einerseits und ihre Kultur andererseits. Wir unterscheiden drei Welten, die die meisten Unternehmen in unterschiedlichen Proportionen miteinander verbinden: (1) die bürokratische Welt mit ihrer Orientierung an Hierarchie, Kontrolle und Vorhersagbarkeit, (2) die Projektwelt mit den Werten Team, Kooperation und Entwicklung, (3) die mit Freiheit, Autonomie und Unsicherheit verbundene Transaktions- oder Gig-Welt. Der richtige Zuschnitt der drei Welten im Unternehmen (wieviel braucht es von welcher Welt?) ebenso wie die Passung der Unternehmenskultur zu den Welten (fördert oder behindert die vorherrschende Kultur die Organisation?) sind wichtig u. a. für Recruiting und Bindung von Talenten – und damit für den Erfolg des Unternehmens.

**Zusatzmaterial online:**

Die englische Übersetzung ist in der Online-Version dieses Artikels (10.1007/s11613-021-00727-2) enthalten.

## Abstract (en)

Covid-19 accelerates virtual forms of global collaboration. This has far-reaching consequences for the structure and processes of organizations on the one hand and their culture on the other. We distinguish between three worlds, which most companies connect in different proportions. The bureaucratic world with its orientation towards hierarchy, control and predictability, the project world with the values team, cooperation and development, and the transaction or gig-world associated with freedom, autonomy and uncertainty. We explain that the right fit of those three worlds in the company (how much of which world does it need?) as well as the fit of the corporate culture to the worlds (does the prevailing culture promote or hinder the organization?) are both important for recruiting and retaining talent—and thus for the success of the company.

## Einführung

Die Pandemie hatte eine außerordentliche und unerwartete Konsequenz: Die Menschen haben sich durch Zoom, Hangout und Teams daran gewöhnt und finden es völlig normal, virtuell zusammenzuarbeiten. Diese Plattformen erleichtern alle Arten der Wissensarbeit, wenn Menschen sich nicht persönlich treffen können. Noch wichtiger ist dabei, dass Menschen und Organisationen heute eine globale Reichweite haben. Vor etwa sechzig Jahren beschrieb Marshall McLuhan ein im Entstehen begriffenes „globales Dorf“ (McLuhan 1962), und er wurde vielfach verspottet, weil er seine Prophezeiung auf das Fernsehen stützte. Heute wissen wir, dass es dazu mehr benötigt als das Fernsehen, nämlich genau das, was diese Plattformen bieten: die Fähigkeit, die ganze Welt zu erreichen, dabei auf Wissen und Ressourcen zuzugreifen und relevante Aufgaben zu bearbeiten. Damit können Organisationen überall Talente finden und einstellen, wenn sie diese Plattformen zur Koordinierung der Arbeit nutzen. Dies beeinflusst, wie Organisationen Talente finden, wie Menschen ihre Karrieren aufbauen und wie globale Arbeitsmärkte funktionieren. Die Auswirkungen sind vielfältig.

In diesem Beitrag konzentrieren wir uns darauf, wie dadurch die Kultur, die Struktur und die Prozesse von Organisationen beeinflusst werden. Wir unterscheiden zu diesem Zweck drei organisatorische und kulturelle Paradigmen, die wir die *bürokratische Welt* (B-Welt), die *Projektwelt* (P-Welt) und die *Transaktionswelt* (T-Welt) nennen (vgl. Tab. 1, 2 und 3). Im globalen Dorf treten die beiden letztgenannten Welten zunehmend in den Vordergrund. Führungskräfte stehen vor der Herausforderung, diese drei Welten trotz ihrer kulturellen und strukturellen Unterschiede zusammenzuhalten. Diese Herausforderung setzt einen Rahmen für das Personalmanagement im kommenden Jahrzehnt. Sie schafft auch die Voraussetzungen für die vor uns liegende Organisationsentwicklungsarbeit. Im Folgenden charakterisieren wir die drei Welten.

**Table 1:** 

B‑Welt	P‑Welt	T‑Welt
Buchhalter	Handwerker einer Dombauhütte	Tagelöhner, Heimarbeiter

**Table 2:** 

B‑Welt	P‑Welt	T‑Welt
Großraumbüro	Co-Working Space	Ungebunden

**Table 3:** 

B‑Welt	P‑Welt	T‑Welt
Angestellte	Talente	Auftragnehmer

In der *bürokratischen Welt* (B-Welt) liegt das Hauptaugenmerk auf den Rollen, die Menschen in einem Unternehmen einnehmen. Die Menschen identifizieren sich in erster Linie mit der Organisation und konzentrieren sich auf ihre eigene Position sowie ihre Beziehungen in der Hierarchie. Die Schlagworte lauten *Hierarchie, Kontrolle* und *Vorhersehbarkeit*. Die Menschen sind loyal. Ihre Arbeit dient der Aufrechterhaltung der Organisation sowie der Förderung ihrer Perspektiven innerhalb der Organisation. Dies ist die überkommene und etablierte Welt, auch wenn ihre Elemente in den letzten Jahrzehnten dadurch erheblich geschwächt werden, dass eine Vielzahl von Organisationen andere kulturelle Aspekte integrieren und dass immer weniger Menschen erwarten, innerhalb einer einzigen Organisation eine Karriere aufzubauen.

Die *transaktionale Welt* (T-Welt, auch: Gig-Welt) ersetzt Hierarchien durch Märkte und Rollen durch Jobs. Sie geht damit noch weiter, als wir es aus holokratischen oder soziokratischen Organisationen kennen. Dies ist der Schauplatz des freiberuflichen Arbeitsmarktes, wo Angestellte zu Auftragnehmern und Freelancern werden. Die Leitworte lauten hier *Freiheit* und *Autonomie* – erkauft durch *Unsicherheit* – sowie *Markttransaktionen*. Diese Kultur wird von der Online-Kommunikation und ihrer globalen Reichweite getragen. Unternehmen veröffentlichen ihre Arbeitsangebote auf digitalen Markplätzen bzw. Plattformen: Fotos machen, Daten eingeben, ein Software-Programm schreiben, Möbel transportieren, Gartenarbeit verrichten, usw. Diese Art der freiberuflichen Tätigkeit gestaltet den Arbeitsmarkt radikal um. Forbes schätzte, dass im Jahr 2018 fast ein Drittel der Arbeitenden freiberuflich tätig war und etwa 1 Mrd. Stunden arbeitete, und damit werden die Auswirkungen dieses Sektors wegen seiner globalen Reichweite noch unterschätzt: Menschen in Indien, Taiwan oder auf den Philippinen können Daten eingeben, Informationen aus Websites extrahieren, Websites erstellen oder Adressen bei LinkedIn heraussuchen (Forbes 2018).

Die *Projektwelt* (P-Welt) beruht darauf, die besten Talente weltweit zu gewinnen, zu fördern und einzusetzen. Die Menschen in der Projektwelt sichern ihre Identität, im Gegensatz zu denen in der bürokratischen Welt, mehr durch ihre Arbeit als durch die Position, die sie einnehmen. In der transaktionalen oder *Gig-Welt* sind Menschen oft austauschbar, weil ihr Know-how weit verbreitet oder unspezifisch ist. Sie sind ein tatsächliches oder virtuelles „Paar Hände“. In der *Projektwelt* ist eine Person dagegen nicht so leicht zu ersetzen, weil sie unverwechselbare Beiträge zur Projektarbeit leistet, indem sie ihr Faktenwissen, ihre spezifischen Fähigkeiten und Erfahrungen sowie ihr persönliches Netzwerk kombiniert. In der P‑Welt ist das „Humankapital“ daher genauso wichtig wie das Finanzkapital. In diesem Umfeld wollen Führungskräfte die Bedingungen schaffen, die es talentierten Menschen ermöglichen, ihre beste Arbeit zu leisten. Das bedeutet, die jeweilige Situation der Mitarbeiter zu berücksichtigen, ihre Arbeit von zu Hause aus zu erleichtern, ihnen die besten Online-Tools und Hardware zur Verfügung zu stellen, die sie für ihre Arbeit benötigen, ihr Lernen zu unterstützen und hervorragende Projektleiter einzusetzen, die die Arbeit über Disziplinen, Ländergrenzen, Zeitzonen und Persönlichkeiten hinweg effektiv orchestrieren können. Die Schlagworte lauten *Kooperation, Team* und *Entwicklung*. Führungskräfte konzentrieren sich weniger darauf, loyale Anhänger oder Organisationsangehörige (*Organisational Citizens*) zu kultivieren, sondern vielmehr darauf, Menschen für die Arbeit einzusetzen, die diese am besten beherrschen. Führungskräfte ermöglichen Produktivität, anstatt sie zu lenken. In dieser Welt werden die Menschen weniger durch ihren Platz in der Hierarchie, sondern vielmehr durch die Qualität ihrer Arbeit und deren Übereinstimmung mit ihren Talenten und Interessen zufriedengestellt. Die Arbeit hält die Organisation zusammen und nicht die Organisation die Arbeit. An der Basis der *Projektwelt* organisieren und verwalten sich Menschen und Teams selbst. Menschen werden eher befähigt als beaufsichtigt, während die Arbeit selbst den Rhythmus des Arbeitsprozesses und die Art und Weise bestimmt, wie Spezialisten zusammenarbeiten. Die mit dem agilen Arbeiten verbundenen Konzepte, die zuerst im Bereich der Software-Entwicklung aufkamen, sind hier am relevantesten.

## Die multi-kulturelle Landschaft: Das Drei-Welten-Modell

Diese drei verschiedenen Welten bringen eigene Organisationskulturen hervor und werden wiederum von diesen verschiedenen Organisationskulturen getragen (Schein 2003), wie Tab. 4 zeigt.

**Table 4:** 

Kulturdimension	Bürokratische Welt	Projekt-Welt	Transaktions-Welt
Zeit	Uhr	Ereignis	Termin
Raum	Hierarchie	Team	Marktplatz
Rahmen	Rolle	Spezialgebiet	Job
Beziehungsmuster	Anpassung	Interaktion	Transaktion
Quelle der Identität	Organisation	Arbeit	Tauschakt
Quelle der Autorität	Chef	Geleistete Arbeit	Vertrag
Ein Tabu	Nicht überraschen	Nicht enttäuschen	Nicht betrügen
Eine Norm	Loyalität	Verfügbarkeit	Verlässlichkeit
Ein Wert	Kontrolle	Zusammenarbeit	Erledigung

Das Ergebnis ist eine multikulturelle Landschaft innerhalb einer einzigen Organisation: Nach dem Drei-Welten-Modell ist die *B‑Welt* für die Aufrechterhaltung der Organisation verantwortlich, übernimmt also eine stabilisierende und führende Aufgabe. Die multikulturelle Herausforderung besteht für die Menschen der B‑Welt darin, alle drei „Sprachen“ zu beherrschen, ohne sie zu verwirren. Eine Führungskraft der B‑Welt kann z. B. nicht erwarten, dass Fachleute der P‑Welt aus Loyalität auf Weisungen reagieren. In ähnlicher Weise können HR-Fachleute zwar von Managern der B‑Welt erwarten, dass sie sich an die Unternehmensrichtlinien halten, auch wenn letztere ihre Relevanz nicht verstehen, andererseits werden Fachleute der P‑Welt Richtlinien nur dann befolgen, wenn sie deren Logik und Bedeutung verstehen.

Der *P‑Welt* obliegt es demgegenüber, die Änderungs- und Anpassungsfähigkeit eines Unternehmens im globalen Wettbewerb sicherzustellen; ohne sie droht Erstarrung in der Routine, und die Existenz der Organisation steht zur Disposition. Die P‑Welt hat daher die wichtige Aufgabe, die aktuelle Positionierung durch die B‑Welt immer wieder in Frage zu stellen, konstruktiv zu irritieren und sogar, ohne Angst vor Kannibalisierung, funktionierende Geschäftsmodelle durch Innovation von innen anzugreifen. Die große Herausforderung für Menschen aus der P‑Welt, die sich vor allem über die Inhalte ihrer Arbeit definieren und motivieren und die kaum ein originäres Interesse daran haben, eine Organisation in Bewegung zu setzen, besteht darin, einen Weg zu finden, sich mit der Kontrolle durch die B‑Welt zu arrangieren und die Sprache der B‑Welt zu lernen, um dort gehört zu werden: Denn wenn sie ihre dynamisierende Aufgabe im Organisationskontext nicht ernst nehmen und nicht ausfüllen, gefährden sie die Basis, die ihnen ihre „eigentliche“ Arbeit erst ermöglicht.

Die T‑Welt vergrößert schließlich die globale Reichweite eines Unternehmens in Bezug auf standardisierbare Arbeiten und ermöglicht gleichzeitig Einsparungseffekte, die lokal nicht erzielbar wären. So unterstützt die T‑Welt die globale Konkurrenzfähigkeit von Unternehmen.

Die kulturellen Unterschiede zwischen den drei Welten, die in der Tab. 4 aufgeführt sind, seien beispielhaft anhand der Kulturdimension „Zeit“ erläutert:

Für die B‑Welt ist tatsächlich die mit der Uhr gemessene Zeit die Grundlage für Denken, Planung und Kontrolle: Wann (Datum, Uhrzeit) wird mit einer Arbeit begonnen? Wann wird sie beendet? Wann wird etwas geliefert? Wie viele Personen arbeiten gleichzeitig wie lange an einer Aufgabe? Die Kosten stehen in einer linearen Beziehung zur Zeit und lassen sich mithilfe der Grundrechenarten jederzeit einfach bestimmen; Überraschungen sind ausgeschlossen und buchstäblich undenkbar. In der T‑Welt steht hingegen der Termin im Fokus, an dem oder bis zu dem spätestens eine Leistung erbracht werden muss. Der Preis für eine Leistung wird meist im Voraus vereinbart und ist fest; der durch den Auftragnehmer für die Erledigung tatsächlich aufgewendete Zeitaufwand spielt keine Rolle. Noch anders in der P‑Welt: Ein Projekt hat einen Anfang und ein Ende. Zwischen diesen Endpunkten wird der Fortschritt anhand der Erreichung von Zwischenzielen („Meilensteinen“) gemessen, mit denen meist auch bestimmte, im Projektverlauf zu produzierenden Assets einer erwarteten Qualität verbunden sind. Da ein Projekt immer ein einmaliges, nicht wiederholbares Vorhaben darstellt, sind zeitliche Schätzungen (Aufwand, Dauer) generell schwierig und unzuverlässig.

Diese kulturellen Unterschiede führen dazu, dass derselbe Begriff in den drei Kulturen mit jeweils verschiedener Bedeutung verwendet wird und mit verschiedenen Konnotationen verbunden ist. Gerade an den Schnittstellen zwischen den unterschiedlichen Welten führt der spezifische Sprachgebrauch immer wieder zu eklatanten Missverständnissen, sodass es in diesem Zusammenhang gerechtfertigt ist, von verschiedenen „Sprachen“ zu sprechen, deren Verwendung gelernt werden muss.

## Die multi-kulturelle Landschaft: Herausforderungen

Bei Entwicklung und Management dieser multikulturellen Welt stehen Organisationen vor drei großen Herausforderungen:

- Das Engagement von Projektwelt-Beschäftigten sichern, auch wenn diese sich auf die Arbeit und nicht auf die Organisation konzentrieren. Es wird einen globalen Wettbewerb um Talente geben. Menschen, die von zu Hause arbeiten können und die die größte Freude aus der Zusammenarbeit mit ihren Kollegen sowie aus ihren Leistungen ziehen, werden der Organisation gegenüber nur dann loyal sein, wenn sie ihren Bedürfnissen entgegenkommt.
- Menschen aus der bürokratischen Welt befähigen, Menschen in der Projektwelt zu führen, wenn keine hierarchische Autorität verfügbar ist. Dies ähnelt der Herausforderung, kreative Menschen zu managen.
- Sicherstellen, dass Menschen in der transaktionalen Welt sich nicht entmenschlicht und missachtet fühlen. Auch wenn die Menschen in der Gig-Welt für die Organisation unsichtbar sein mögen, so können sie doch, vermittelt durch soziale Medien, das Image der Organisation nachhaltig beschädigen, wenn sie sich von ihr schlecht behandelt fühlen.

Die relative Relevanz dieser Herausforderungen hängt davon ab, wie wichtig jede dieser Welten für das Gesamtdesign bzw. die Architektur der Organisation ist.

## Die Drei Welten in der Beratungsarbeit: Struktur vs. Kultur

Für eine wirksame Beratungsarbeit ist es wichtig, beide Ebenen herauszuarbeiten, zu betrachten und miteinander in Beziehung zu setzen: die Organisationsstruktur (Geometrie, Aufbauorganisation, Prozesse, Rollen etc.) einerseits und die in einem Unternehmen parallel existierenden Organisationskulturen andererseits. Auf der Strukturebene geht es u. a. um den richtigen Zuschnitt: Wieviel braucht ein Unternehmen von welcher Welt, um effektiv und effizient arbeiten zu können? Auf der Kulturebene stellt sich die Frage, inwiefern die aktuell vorherrschende Kultur die jeweilige Organisationsform unterstützt oder ihr möglicherweise – oft unbemerkt – entgegenwirkt.

### Organisationsgeometrien

**Figure 1:**
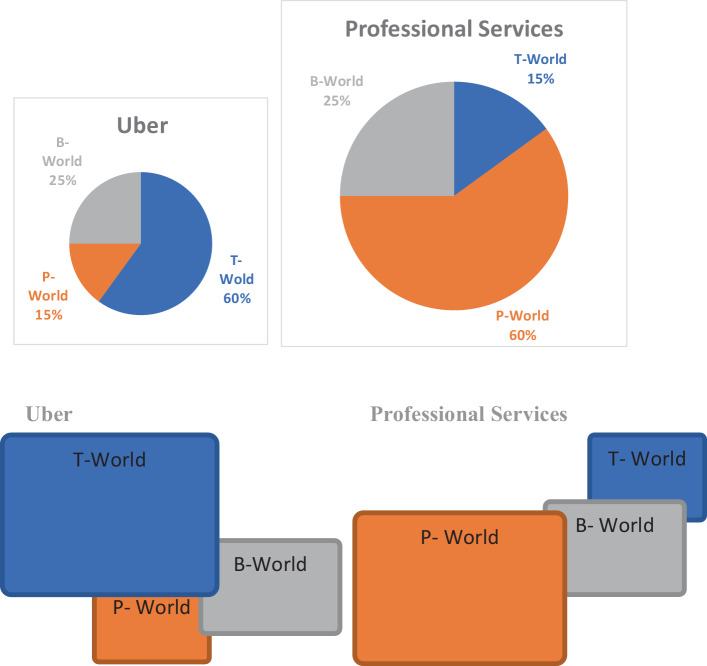

Den Entwurf einer passenden Organisationsgeometrie können wir uns vereinfacht und schematisch wie folgt vorstellen. Auf der Grundlage der zu leistenden Arbeit und der Zuordnung der Aufgaben zu den drei verschiedenen Welten wählt ein Unternehmen seine Organisations-Geometrie aus, indem es die relativen Proportionen der einzelnen Organisationstypen plant (Abb. 1).

In diesem Diagramm besitzt z. B. ein professioneller Dienstleister eine kleine B‑Welt, die als operativer Kern fungiert, und eine verhältnismäßig größere P‑Welt, um die erforderliche Wissensarbeit zu leisten. Im Gegensatz verwendet eine Firma wie Uber eine proportional größere T‑Welt – die treibende Kraft – und eine kleine P‑Welt, um ihre Technologieplattform zu entwickeln und zu pflegen. Die Geometrie einer Organisation hängt also vom Geschäftsmodell ab.

### Organisationskulturen

Fokussieren wir uns auf die Kulturebene und die damit verbundenen Aspekte der drei Welten, dann können wir untersuchen, inwiefern ein Unternehmen unbemerkt mit der einen Kultur arbeitet, obwohl es eine andere Organisationsform favorisiert. Die Diskrepanz von Kultur und Organisationsstruktur kann das Erreichen der Unternehmensziele signifikant behindern. Wird eine Diskrepanz diagnostiziert, kann ihre Analyse spannende Implikationen beinhalten und zu vertieften Erkenntnissen bzw. Einsichten führen. Sie bildet dann oft die Grundlage für einen neuen Umgang mit den Strukturen. Wir illustrieren dies mit zwei Beispielen, um den oft verborgenen und übersehenen Kulturaspekt herauszuarbeiten.

#### Beispiel 1: Bürokratie schlägt Projektarbeit im Logistikkonzern

Das folgende Beispiel zeigt, wie das Modell zur Lösung von Herausforderungen beiträgt: In der Vergangenheit blieben die Ergebnisse wichtiger Projekte eines Logistikkonzerns weit hinter den Erwartungen zurück. Eine Analyse mit dem HR-Leiter kam zu dem Ergebnis, dass unbemerkt die Dominanz der B‑Welt-Logik die Projektarbeit behinderte. Dies lag an folgenden Punkten:

- Karriere wurde durch Ausübung von Führung in Linienfunktion begünstigt, nicht durch erfolgreiche Projektarbeit.
- Führungskräfte, die längere Zeit in einem Projekt gearbeitet haben, hatten im Anschluss daran keinen Anspruch auf ihre vorherige Position.
- Projekte kamen zum normalen Arbeitspensum hinzu; es blieben nicht genug Ressourcen für Projektarbeit.
- Weiche Faktoren wie Teamarbeit und Kooperation wurden für irrelevant erklärt.
- Leistungsstarke Führungskräfte hielten sich von Projekten fern oder engagierten sich nur pro-forma.
- Projekte wurden von weniger erfolgreichen Führungskräften übernommen.

Alle Punkte sprechen für die kulturelle Dominanz der B‑Welt. Letztlich werden Hierarchie und die Rolle höher bewertet als Teamarbeit und Entwicklung. Um die Projektarbeit zu stärken, wurden folgende Schritte unternommen:

- Angesprochen wurden besonders leistungsstarke Führungskräfte.
- Es wurden Ressourcen für die Entwicklung des Projektteams zur Verfügung gestellt.
- Führungskräften wurde die Rückkehr auf die gleiche bzw. nächsthöhere Position wie vor Projektbeginn vertraglich zugesichert.
- Die freiwerdenden Führungspositionen wurden mit Vertretern besetzt, deren Entwicklung für die Zeit nach dem Projekt ebenfalls definiert wurde.

Diese Maßnahmen trugen zur enormen Stärkung der P‑Welt bei. Projekte wurden nun wesentlich öfter als früher erfolgreich durchgeführt.

#### Beispiel 2: Zuviel Gig und zu wenig Projekt im Startup

Ein junger, international agierender Anbieter von Sprachprogrammen war konsequent nach dem transaktionalen Modell aufgebaut. Überall auf der Welt wurden Übersetzer angeworben, die Texte von einer in eine andere Sprache übersetzten. Gleichzeitig arbeiteten Programmierer daran, die vorhandenen Übersetzungen in die Plattform zu integrieren und deren Leistungsfähigkeit durch künstliche Intelligenz weiterzuentwickeln. Nach erfolgreichem Wachstum war die Firma mit folgenden Problemen konfrontiert: Das Ausmaß an Doppelarbeit stieg rasant an, und die Kontrolleinheit war mit der Korrektur der Übersetzungen und der Algorithmen überfordert. Mit wachsender Komplexität stieg der Anteil der engagierten Übersetzer, die sich nicht an das Regelwerk hielten und die Firma rasch wieder verließen. In der Diskussion mit dem Leitungsgremium wurde zunächst erwogen, die B‑Welt zu stärken: formalisiertes Recruiting und Ausarbeitung von Standardisierungstools standen im Zentrum der Überlegungen. Die Auseinandersetzung mit dem Drei-Welten-Modell führte zur naheliegenden und dennoch überraschenden Lösung: Statt auf eine reine Transaktionswelt zu setzen, wurde der Projektteam-Gedanke gestärkt. Es wurden internationale Teams gebildet, in denen sich die Freelancer gegenseitig berieten, Regeln entwickelten und sogar Recruiting im eigenen Netzwerk vornahmen. Die Bildung stabiler Gruppen senkte das Ausmaß von Doppelarbeit und reduzierte die Fehler mit Hilfe eines eingeführten Peer-Review-Verfahrens. An die Rolle des Scrum Masters angelehnt, wurden den Teams besonders ausgebildete Koordinatoren zur Verfügung gestellt. Kooperationszeiten wurden darüber hinaus als Arbeitszeit vergütet.

## Empfehlungen für die Unternehmenspraxis

Das Denkmodell der drei Welten legt Führungskräften und Teams nahe, sich mit den folgenden Fragen zu beschäftigen:

1. Ist-Analyse Ihrer Organisationsgeometrie: In welchem Verhältnis stehen in Ihrem Unternehmen die drei Welten heute zueinander? Wie sind die Aufgaben heute verteilt? Passen Organisationsgeometrie und Aufgabenverteilung zu den Herausforderungen, denen sich Ihre Organisation gegenübersieht?
2. Ist-Analyse Ihrer Organisationskultur: Überlegen Sie anhand der Tabelle, welche Kulturelemente in Ihrem Unternehmen vorherrschen und ob diese die jeweiligen Welten unterstützen oder behindern.
3. Diskutieren Sie, welche Proportionen der drei Welten Sie in Zukunft brauchen.
4. Welche Aufgaben gehören zukünftig in welche Teilwelten?
5. Wie und in welchen Schritten gelangen Sie vom Status Quo zur richtigen Größe, zu den passenden Proportionen der verschiedenen Welten und zur neuen Aufgabenverteilung?
6. Welche HR-, Rekrutierungs- und Entwicklungsinitiativen sollten in Ihrer Firma jetzt ergriffen werden, um diesen Wandel zu vollziehen?
7. Wie pflegen Sie Talente für die B‑ und die P‑Welt, und welche unterschiedlichen Personalstrategien brauchen Sie für die B‑ und die P‑Welt?

## Supplementary Information




