# Supplementary material for: Three cultural and organizational worlds
Source: Organisationsberat Superv Coach. 2021 Nov 3;28(4):543–52. [Article in German] doi: 10.1007/s11613-021-00727-2 (PMC8564589; doi:10.1007/s11613-021-00727-2)
Supplement: Supplementary file 1 [file 11613_2021_727_MOESM1_ESM.doc]

**Three cultural and organizational worlds**

**Larry Hirschhorn, Thomas Giernalczyk, Martin Holle, Mathias Lohmer, Markus Zimmermann**

Submitted: 26.2.2021 / Accepted: 23.5.2021

**Abstract** Covid-19 accelerates virtual forms of global collaboration. This has far-reaching consequences for the structure and processes of organizations on the one hand and their culture on the other. We distinguish between three worlds, which most companies connect in different proportions. The bureaucratic world with its orientation towards hierarchy, control and predictability, the project world with the values team, cooperation and development, and the transaction or gig-world associated with freedom, autonomy, and uncertainty. We explain that the right fit of those three worlds in the company (how much of which world does it need?) as well as the fit of the corporate culture to the worlds (does the prevailing culture promote or hinder the organization?) are both important for recruiting and retaining talent – and thus, for the success of the company.

**Keywords** Covid-19, virtual collaboration, bureaucratic world, project world, transaction or gig-world, recruiting, corporate design, human resources

**Drei kulturelle und organisatorische Welten**

**Zusammenfassung** Covid-19 beschleunigt virtuelle Formen der weltweiten Zusammenarbeit. Das hat weitreichende Konsequenzen für Struktur und Prozesse von Organisationen einerseits und ihre Kultur andererseits. Wir unterscheiden drei Welten, die die meisten Unternehmen in unterschiedlichen Proportionen miteinander verbinden: (1) die bürokratische Welt mit ihrer Orientierung an Hierarchie, Kontrolle und Vorhersagbarkeit, (2) die Projektwelt mit den Werten Team, Kooperation und Entwicklung, (3) die mit Freiheit, Autonomie und Unsicherheit verbundene Transaktions- oder Gig-Welt. Der richtige Zuschnitt der drei Welten im Unternehmen (wieviel braucht es von welcher Welt?) ebenso wie die Passung der Unternehmenskultur zu den Welten (fördert oder behindert die vorherrschende Kultur die Organisation?) sind wichtig u.a. für Recruiting und Bindung von Talenten – und damit für den Erfolg des Unternehmens.

**Schlüsselwörter** Covid-19, virtuelle Zusammenarbeit, bürokratische Welt, Projektwelt, Transaktions-Welt, Personalentwicklung

Prof. Dr. T. Giernalczyk

Universität der Bundeswehr München

M19-Manufaktur für Organisationsberatung GmbH, Bauerstraße 19, D-80796 München

E-Mail: [Thomas.giernalczyk@unibw.de](mailto:Thomas.giernalczyk@unibw.de)

L. Hirschhorn, Ph.D.

Park Square, 31 Saint James Avenue, Suite 720, Boston, MA 02116, USA

E-Mail: hirschhorn@cfar.com

M. Holle

Grünenthal 30, D-52072 Aachen

E-Mail: [mh@martin-holle.de](mailto:mh@martin-holle.de)

Dr. phil. M. Lohmer

Feilitzschstraße 36, D-80802 München

E-Mail: [mathias.lohmer@m19-organisationsberatung.de](mailto:mathias.lohmer@m19-organisationsberatung.de)

M. Zimmermann

Bauerstraße 19, D-80796 München

E-Mail: [markus.zimmermann@m19-organisationsberatung.de](mailto:markus.zimmermann@m19-organisationsberatung.de)

**1 Introduction**

The pandemic had an extraordinary and unexpected consequence: People got used to it through zoom, hangout and teams and find it completely normal to work together virtually. These platforms facilitate all kinds of knowledge work when people cannot meet in person. More importantly, people and organizations today have a global reach. About sixty years ago, Marshall McLuhan described an emerging “global village” (McLuhan 1962) and he was often mocked because he based his prophecy on television. Today we know that it takes more than television to do this, namely exactly what these platforms offer: the ability to reach the whole world, to access knowledge and resources and to work on relevant tasks. So, organizations everywhere can find and hire talent when they use these platforms to coordinate work. This influences how organizations find talent, how people build their careers and how global labor markets work. The implications are many and varied. In this paper we focus on how this affects the culture, structure, and processes of organizations.

To this end, we distinguish three organizational and cultural paradigms, which we call the *bureaucratic world* (B world), the *project world* (P world) and the *transaction world* (T world) (cf. tab. 1-3). In the global village, the latter two worlds are increasingly coming to the fore. Managers face the challenge of keeping these three worlds together despite their cultural and structural differences. This challenge sets a framework for human resource management in the coming decade. It also creates the conditions for the organizational development work ahead. In the following we characterize the three worlds.

| *B-World* | *P-World* | *T-World* |
| --- | --- | --- |
| Accountant | Craftsman of a cathedral building hut | day laborer, home worker |

**Tab. 1** A historical embodiment

In the *bureaucratic world* (B world), the main focus is on the roles that people play in a company. People primarily identify with the organization and focus on their own position and their relationships in the hierarchy. The keywords are *hierarchy, control, and predictability*. People are loyal. Their work serves to maintain the organization and to promote their perspectives within the organization. This is the traditional and established world, although its elements have been considerably weakened in recent decades by the fact that many organizations integrate other cultural aspects, and that fewer and fewer people expect to build a career within a single organization

| *B-World* | *P-World* | *T-World* |
| --- | --- | --- |
| Open-plan office | Co-Working Space | Unattached |

**Tab. 2** Natural environment

The *transactional world* (T world, also: gig world) replaces hierarchies with markets and roles with jobs. This is the arena of the freelance labor market, where employees become contractors and freelancers. The key words here are *freedom* and *autonomy* – bought by *insecurity* – and *market transactions*. This culture is supported by online communication and its global reach. Companies publish their job offers on digital marketplaces or platforms: Taking photos, entering data, writing a software program, transporting furniture, doing gardening, etc. This type of freelance work is radically transforming the job market. Forbes estimated that in 2018, almost one-third of workers were freelancers and worked around 1 billion hours, underestimating the impact of this sector because of its global reach: people in India, Taiwan or the Philippines can enter data, extract information from websites, create websites or look up addresses on LinkedIn (Pofeldt 2018).

| *B-World* | *P-World* | *T-World* |
| --- | --- | --- |
| Employee | Talents | Contractor |

**Tab. 3** Relationship to staff

The *Project World* (P-World) is based on attracting, promoting, and deploying the best talents worldwide. Unlike those in the bureaucratic world, people in the Project World secure their identity more through their work than through the position they occupy. In the transactional or *Gig-world,* people are often interchangeable because their know-how is widespread or unspecific. They are an actual or virtual “pair of hands”. In the *project world*, on the other hand, a person is not so easy to replace because they make distinctive contributions to project work by combining their factual knowledge, their specific skills and experience, and their personal network. In the P-world, therefore, “human capital” is just as important as financial capital. In this environment, managers want to create the conditions that enable talented people to do their best work. This means considering the respective situation of the employees, their Facilitate work from home, provide them with the best online tools and hardware they need for their work, support their learning, and employ excellent project managers who can effectively orchestrate work across disciplines, countries, time zones, and personalities. The keywords are *cooperation, team* and *development*. Leaders focus less on cultivating loyal followers or organizational citizens and more on employing people who are best at their jobs. Leaders enable productivity rather than directing it. In this world, people are satisfied not so much by their place in the hierarchy, but rather by the quality of their work and its alignment with their talents and interests. Work holds the organization together and not the organization holds the work. At the base of the project world, people and teams organize and manage themselves. People are empowered rather than supervised, while the work itself determines the rhythm of the work process and the way specialists work together. The concepts associated with agile working, which first appeared in the field of software development, are most relevant here.

**2 The multi-cultural landscape: the three-world model**

These three different worlds produce their own organizational cultures and are in turn supported by these different organizational cultures (Schein 1999), as shown in tab. 4.

| **Cultural Dimension** | **Bureaucratic world** | **Project World** | **Transaction World** |
| --- | --- | --- | --- |
| *Time* | Clock | Event | Date |
| *Space* | Hierarchy | Team | Marketplace |
| *Frame* | Role | Specialty | Job |
| *Relationship pattern* | Adaptation | Interaction | Transaction |
| *Source of identity* | Organization | Work | act of exchange act |
| *Source of authority* | Boss | work performed | Contract |
| *A Taboo* | Do not surprise | Do not disappoint | Do not cheat |
| *A Norm* | Loyalty | Availability | Reliability |
| *A Value* | Control | Collaboration | Dispatch |

**Tab. 4** Organizational cultures

The result is a multicultural landscape within a single organization: According to the three-world model, the *B-world* is responsible for maintaining the organization, i.e., it assumes a stabilizing and leading role. The multicultural challenge for the people in B-World is to master all three “languages” without confusing them. For example, a B-world leader cannot expect P-world professionals to respond to instructions out of loyalty. Similarly, while HR professionals can expect B-world managers to follow corporate policies even if the latter do not understand their relevance, P-world professionals will only follow policies if they understand their logic and meaning.

The *P-World*, on the other hand, is responsible for ensuring the ability of a company to change and adapt in global competition; without it, routine is at risk of becoming rigid and the existence of the organization is at stake. The P-World therefore has the important task of constantly questioning the current positioning by the B-World, constructively irritating and even, without fear of cannibalization, attacking functioning business models through innovation from within. The big challenge for people from the P-World, who define and motivate themselves primarily through the content of their work, and who have hardly any original interest in setting an organization in motion, is to find a way to come to terms with the control by the B-World and to learn the language of the B-World in order to be heard there: Because if they do not take their dynamizing task in the organizational context seriously and do not complete it, they endanger the basis that enables them to do their “real” work.

Finally, the *T-World* increases a company's global reach in terms of work that can be standardized and at the same time enables savings effects that would not be achievable locally. Thus, T-World supports the global competitiveness of companies.

The cultural differences between the three worlds, which are listed in the table, are exemplified by the cultural dimension “time”:

For the B-world, the time measured by the clock is indeed the basis for thinking, planning, and controlling: When (date, time) is a work started? When is it finished? When is something delivered? How many people work simultaneously on a task and for how long? Costs are linearly related to time and can be easily determined at any time using basic arithmetic; surprises are excluded and literally unthinkable. In the G-World, on the other hand, the focus is on the date on or by which a service must be provided at the latest. The price for a service is usually agreed in advance and is fixed; the time actually spent by the contractor on the completion of the work is irrelevant. Even different in the P-world: A project has a beginning and an end. Between these end points, progress is measured by the achievement of intermediate goals (“milestones”), which are usually associated with certain assets of expected quality to be produced during the project. Since a project is always a one-time, non-repeatable undertaking, time estimates (effort, duration) are generally difficult and unreliable.

These cultural differences lead to the fact that one and the same term is used in the three cultures, each with a different meaning and associated with different connotations. It is precisely at the interfaces between the different worlds that the specific use of language repeatedly leads to blatant misunderstandings, so that in this context it is justified to speak of different “languages” whose use must be learned.

**3 The multi-cultural landscape: challenges**

Organizations face three major challenges in developing and managing this multicultural world.

- Ensure the commitment of Project World employees, even if they are focused on the work and not on the organization. There will be global competition for talent. People who can work from home and who derive the greatest pleasure from working with their colleagues and from their achievements will only be loyal to the organization if it meets their needs.
- Empowering people from the bureaucratic world to lead people in the project world when hierarchical authority is not available. This is like the challenge of managing creative people.
- To ensure that people in the transactional world do not feel dehumanized and disregarded. Even though people in the gig world may be invisible to the organization, they can, mediated by social media, cause lasting damage to the image of the organization if they feel mistreated by it.

The relative relevance of these challenges depends on how important each of these worlds is to the overall design or architecture of the organization.

**4 The three worlds in consulting work: structure vs. culture**

For effective consulting work, it is important to work out, consider and relate both levels: the organizational structure (geometry, organizational structure, processes, roles, etc.) on the one hand and the organizational cultures that exist in parallel in a company on the other. On the structural level, it is important to find the right balance: How much of which world does a company need to work effectively and efficiently? On the cultural level, the question arises to what extent the currently prevailing culture supports the respective organizational form or possibly - often unnoticed - counteracts it.

**4.1** **Organization Geometries**

We can imagine the design of a suitable organizational geometry in a simplified and schematic way as follows. Based on the work to be done and the assignment of tasks to the three different worlds, a company selects its organizational geometry by planning the relative proportions of each type of organization (see fig. 1).

In this diagram, a professional service provider, for example, has a small B-world that acts as the operational core and a relatively larger P-world to do the necessary knowledge work. In contrast, a company like Uber uses a proportionally larger T-world – the driving force – and a small P-world to develop and maintain its technology platform. So, the geometry of an organization depends on its business model.

| **Uber** | **Professional Services** |
| --- | --- |
| P- World  B-World  T-World | T- World  B- World  P- World |

**Fig. 1** Proportions of each type of organization

**4.2 Organizational Cultures**

If we focus on the cultural level and the related aspects of the three worlds, we can examine the extent to which a company works unnoticed with one culture even though it favors a different organizational form. The discrepancy between culture and organizational structure can significantly impede the achievement of corporate goals. If a discrepancy is diagnosed, its analysis can have exciting implications and lead to deeper insights. It often forms the basis for a new approach to structures. We illustrate this with two examples to highlight the often hidden and overlooked cultural aspect.

***Example 1: Bureaucracy beats project work in a logistics company***

The following example shows how the model contributes to solving challenges: In the past, the results of important projects of a logistics group have fallen far short of expectations. An analysis with the head of HR concluded that unnoticed the dominance of B-World logic was hindering project work. This was due to the following points:

- Career was favored by exercising leadership in line functions, not by successful project work
- Executives who had worked on a project for a longer period were not entitled to their previous position
- Projects were added to the normal workload; not enough resources remained for project work
- Soft factors like teamwork and cooperation were declared irrelevant
- High-performing managers stayed away from projects or only got involved pro forma
- Projects were taken over by less successful managers

All points speak for the cultural dominance of the B-world. Ultimately, hierarchy and role will be valued higher than teamwork and development. The following steps were taken to strengthen the project work:

- Particularly high-performing managers were addressed
- Resources were made available for the development of the project team.
- Executives were contractually assured that they would return to the same or next higher position as before the start of the project
- The vacant management positions were filled with representatives, whose development for the time after the project was also defined

These measures contributed to the enormous strengthening of the P-World. Projects were now successfully implemented much more often than before.

***Example 2: Too much gig and too little project in the startup***

A young, internationally active provider of language programs was consistently built up according to the transactional model. Translators were recruited all over the world to translate texts from one language into another. At the same time, programmers were working to integrate the existing translations into the platform and to further develop its performance through artificial intelligence. After successful growth, the company faced the following problems: The amount of duplication of work increased rapidly and the control unit was overwhelmed with the correction of the translations and the algorithms. As complexity increased, the percentage of dedicated translators who did not follow the rules and quickly left the company. In the discussion with the management board, the first consideration was to strengthen the B-World: formalized recruiting and the development of standardization tools were at the center of the considerations. The discussion of the three-world model led to the obvious yet surprising solution: instead of focusing on a purely transactional world, the project team idea was strengthened. International teams were formed in which the freelancers advised each other, developed rules and even recruited within their own networks. The formation of stable groups lowered the amount of duplication of work and reduced errors with the help of an introduced peer review process. Based on the role of the Scrum Master, the teams were provided with specially trained coordinators. Cooperation times were also remunerated as working time.

**5 Recommendations for business practice**

The thinking model of the three worlds encourages managers and teams to deal with the following questions:

1. Actual state analysis of your organizational geometry: What is the relationship between the three worlds in your company today? How are the tasks distributed today? Do organizational geometry and task distribution match the challenges facing your organization?
2. Actual analysis of your organizational culture: Use the table to consider which cultural elements predominate in your company and whether they support or hinder the respective worlds.
3. Discuss which proportions of the three worlds you need in the future.
4. Which tasks belong in which sub-worlds in the future?
5. How and in which steps do they move from the status quo to the right size, to the right proportions of the different worlds and to the new distribution of tasks?
6. What HR, recruitment and development initiatives should your company take now to bring about this change?
7. How do you nurture talent for the B and P worlds, and what different HR strategies do you need for the B and P worlds?

**Literatur**

McLuhan, M. (1962). *The Gutenberg Galaxy*. Amsterdam: Amsterdam University Press.

Pofeldt, E. (2018). *Freelance economy continues to roar.* https://www.forbes.com/sites/elainepofeldt/2018/10/31/freelancing-economy-continues-to-roar/#b1763a47df45 (abgerufen am 20.2.2021).

Schein, E. (1999). *The Corporate Culture Survival Guide. Sense and Nonsense of Cultural Change.* San Francisco: Jossey Bass (dt.: *Organisationskultur.* Bergisch Gladbach: EHP, 2003).

**Larry Hirschhorn**, Ph.D., is a principal at CFAR, a management consulting firm with offices in Philadelphia and Boston. He is director of CFAR's Dynamics of Consulting program, a program for experienced trainers and consultants. He holds a PhD in economics from MIT and is the author of numerous books.

**Prof. Dr. Thomas Giernalczyk**, Dipl.-Psych., co-founder and managing director of M19 – Manufaktur für Organisationsberatung and the Institut für Psychodynamische Organisationsberatung München (IPOM). Honorary professor for psychological and therapeutic interventions at the Faculty of Human Sciences of the University of the Federal Armed Forces Munich, coach (DGSv), psychoanalyst and group analyst (MAP/DGPT).

**Martin Holle**, Dipl.-Ing. (RWTH Aachen), senior consultant for information technology and telecommunications (IT/TK), Digitalization expert, Freelance organizational consultant, coach and network partner of M19 – Manufaktur für Organisationsberatung GmbH. More than 30 years of experience as consultant, coach, business analyst and solution architect. Internet: www.martin-holle.de

**Dr. phil. Mathias Lohmer**, Dipl.-Psych., Psychoanalyst (DPV, IPA; DGPT), Consultant, Coach, Supervisor. Partner and shareholder of M19 – Manufaktur für Organi-sationsberatung GmbH, shareholder of „Institut für Psychodynamische Organisati-onsberatung München (IPOM)“. Many years of experience in accompanying change processes, leadership development, cultural change. Internet: www.m19-organisationsberatung.de.

**Markus Zimmermann**, graduate in business administration (FH). Partner at M19 – Manufaktur für Organisationsberatung GmbH. Complementary consultant, free space pilot and Potenzialfor-scher for organizations and humans in change processes. Many years of experience in expert, management and consulting functions in the fields of human resources, leadership & culture development and change management. Internet: www.m19-organisationsberatung.de.
